# Patterns and trends in melanoma mortality in the United States, 1999–2020

**DOI:** 10.1186/s12885-024-12426-z

**Published:** 2024-07-02

**Authors:** Alexander J. Didier, Swamroop V. Nandwani, Dean Watkins, Alan M. Fahoury, Andrew Campbell, Daniel J. Craig, Divya Vijendra, Nancy Parquet

**Affiliations:** 1 The University of Toledo College of Medicine and Life Sciences, 3000 Arlington Ave, Toledo, OH USA; 2 Division of Hematology and Oncology, Department of Medicine, The University of Toledo College of Medicine and Life Sciences, Toledo, OH USA; 3 Division of Dermatology, Department of Medicine, The University of Toledo College of Medicine and Life Sciences, Toledo, OH USA

**Keywords:** Melanoma, United States, Dermoscopy, Morbidity, Immune Checkpoint Inhibitors

## Abstract

**Introduction:**

Melanoma, a deadly form of skin cancer, has witnessed a notable increase in incidence over the past decades. Despite advancements in treatment, it remains a significant cause of cancer mortality. Understanding demographic trends and variations in melanoma mortality is crucial for addressing disparities and implementing effective interventions.

**Methods:**

Using the Centers for Disease Control Wide Ranging Online Data for Epidemiologic Research (CDC WONDER) database, we analyzed melanoma mortality data in the United States from 1999 to 2020. Data were stratified by demographic and regional variables, and age-adjusted mortality rates were calculated. Descriptive analysis was performed and Joinpoint regression analysis was employed to identify temporal trends.

**Results:**

Between 1999 and 2020, there were 184,416 melanoma-related deaths in the United States Overall, the age-adjusted mortality rate declined from 2.7 to 2.0 per 100,000 people at a rate of -1.3% annually, with significant variations across demographic groups and regions. Men, non-Hispanic White individuals, and those aged > 65 experienced higher mortality rates. Non-Hispanic White individuals noted the steepest decrease in AAMR after 2013 at a rate of -6.1% annually. Disparities were seen by geographic density, with rural populations exhibiting higher mortality compared to their urban and suburban counterparts.

**Conclusion:**

The study highlights a significant reduction in melanoma mortality in the U.S. since 2013, potentially attributed to advancements in diagnostic techniques such as dermoscopy and the introduction of immune checkpoint inhibitors. Disparities persist, particularly among rural populations. Targeted interventions focusing on increased screening and education are warranted to further mitigate melanoma mortality and address demographic disparities.

## Introduction

Melanoma is the fifth most common cancer in the United States with a 320% increase in incidence since 1975 [1]. Melanoma is also considered to be one of the deadliest forms of skin cancer, accounting for nearly 75% of skin cancer deaths [2]. The strongest risk factor for melanoma is the duration and amount of UV exposure throughout a person’s lifetime [3]. In addition, family history, genetic background, number of melanocytic nevi, skin color, sun sensitivity, previous history of melanoma, and age are all associated risk factors for developing melanoma [3]. Treatment of melanoma has advanced significantly in the last decade. Prior to the release of checkpoint inhibitors, cytotoxic chemotherapy was the gold standard yet was associated with a low survival rate [4]. However, since the release of novel checkpoint inhibitors since 2011, there has been a significant increase in survival in patients with melanoma with the 5-year relative survival rate approaching 94% when caught early [4]. Despite these newer treatment modalities, melanoma continues to be a significant cause of skin cancer mortality in the United States and globally [4].

National variations and trends exist in melanoma mortality, with various demographic groups affected differently than others. Previous studies have demonstrated increased melanoma incidence and lower survival in rural populations when compared with their urban counterparts [5]. Additionally, non-Hispanic Black populations experience a lower 5-year survival than non-Hispanic White populations, showing that these patients tend to fare worse after their diagnosis [6]. This may be due to the fact that non-Hispanic Black patients have a higher risk of being diagnosed with late-stage melanoma when compared with non-Hispanic White patients [7]. In light of these differences, understanding demographic variations in trends can shed light on concerns regarding disparities seen amongst groups. To date, no population-based study exists assessing demographic differences in trends in melanoma mortality in the United States in the era of checkpoint inhibitors. We analyzed a national database to describe trends seen in melanoma mortality in the United States between 1999 and 2020.

## Methods

### Dataset

The Centers for Disease Control (CDC) Wide-Ranging Online Data for Epidemiologic Research (WONDER) database was queried for mortality statistics with an underlying cause of death of melanoma (International Classification of Diseases, 10th edition (ICD-10) code C43.0.x) between the years 1999–2020. The CDC WONDER database uses mortality statistics collected from death certificates and has been used in other studies assessing cancer mortality in the United States [8, 9]. The data are available publicly online through the CDC WONDER website. This study did not require institutional review board approval because the CDC WONDER is a publicly available database that contains deidentified data. The data used for this study are publicly available at https://wonder.cdc.gov.

We extracted data for melanoma-related deaths between 1999 and 2020. Data were grouped based on demographic and regional variables. We assessed mortality by race (including Hispanic, Non-Hispanic White, Non-Hispanic Black, Non-Hispanic Asian or Pacific Islander, Non-Hispanic American Indian/Native American), geographic density (urban [population ≥ 1 million], suburban [population 50,000–999,999], rural [population < 50,000]) based on the 2013 US census classification, sex (male or female), age (25–44, 45–64, 65 + years), and US census region. Regions were classified into Northeast, Midwest, South, and West, according to the US Census Bureau definitions [10].

### Statistical analysis

Melanoma age-adjusted mortality rates (AAMRs) per 100,000 people were calculated and standardized to the year 2000 United States Population [11]. Joinpoint Regression software (National Cancer Institute, Bethesda, MD) was used to determine temporal trends in AAMR. Annual percent change (APC) and average annual percent change (AAPC) were calculated. Joinpoint regression identifies significant changes in AAMR over time by using a Monte Carlo permutation method to identify an optimal number of joinpoints, which are line segments connecting two data points [12]. Next, the model segments the entire period by those joinpoints and estimates an annual percent change for each segment. APCs were considered increasing or decreasing if the slope describing the change in mortality was significantly different than 0 using 2-tailed t testing. Statistical significance was set at *p* < 0.05.

## Results

Between 1999 and 2020, melanoma led to 184,416 deaths in the United States. The AAMR decreased from 2.7 in 1999 to 2.0 in 2020 with an associated APC of -1.3% (95% CI -1.9 to -0.8). Between 1999 and 2013, the APC rose at 0.2%, then between 2013 and 2017, the APC began to fall at a rate of -6.6%. After 2017, the APC fell at a slower rate of -1.6%.

**Fig. 1:**
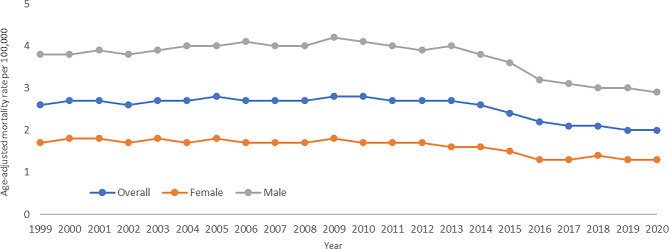
Trends in age-adjusted, melanoma‐related mortality rates stratified by sex in the United States, 1999 to 2020. Overall: 1999–2013 APC 0.2 (95% CI -0.1 to 0.6), 2013–2017 APC − 6.6* (95% CI -8.5 to -2.6), 2017–2020 APC − 1.6 (95% CI -4.2 to 1.9); Female: 1999–2013 APC − 0.3 (95% CI -3.2 to 2.6), 2013–2016 APC − 7.3 (95% CI -10.2 to 3.1), 2016–2020 APC − 0.6 (95% CI -4.4 to 6.5); Male: 1999–2009 APC 0.9* (95% CI 0.5 to 1.8), 2009–2014 APC − 1.5 (95% CI -2.5 to 0.2), 2014–2017 APC − 7.3* (95% CI -8.7 to -5.1), 2017–2020 APC − 1.5 (95% CI -3.8 to 2.2) *Indicates that the annual percentage change (APC) is significantly different from 0 at α = 0.05

In 1999, men had an AAMR of 3.8, over twice as high as that of females who had an AAMR of 1.7 (Fig. 1). For the male cohort, the APC rose at a rate of 0.9% between 1999 and 2009. After 2009, the APC fell at a rate of -1.5% until 2014, where it began to fall at a steeper rate of -7.3%, and then the slope flattened to -1.5% until 2020. For women, the APC decreased at a rate of -0.2% between 1999 and 2011, and then at a rate of -3.5% between 2011 and 2020.

**Fig. 2:**
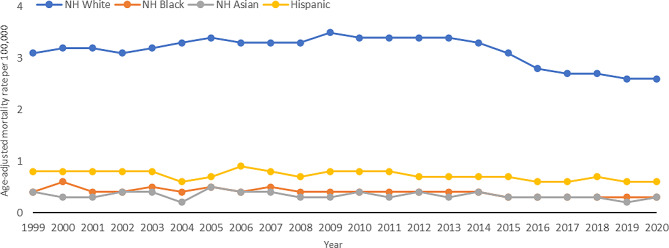
Trends in age-adjusted, melanoma‐related mortality rates stratified by race/ethnicity in the United States, 1999 to 2020. NH White: 1999–2013 APC 0.7* (95% CI 0.4 to 1.1), 2013–2017 APC − 6.1 (-8.2 to 0.5), 2017–2020 APC − 1.3 (95% CI -4.1 to 2.4); NH Black 1999–2020 APC − 2.3* (95% CI -3.4 to -1.2); NH Asian or Pacific Islander: 1999–2020 APC − 1.2 (95 CI -2.7 to 0.4); Hispanic: 1999–2020 APC − 1.2* (95% CI -2.0 to -0.5) *Indicates that the annual percentage change (APC) is significantly different from 0 at α = 0.05

Between 1999 and 2020, Non-Hispanic (NH) White individuals demonstrated the highest AAMR at 3.2, followed by Hispanic individuals at 0.7, NH African American at 0.4, and NH Asian or Pacific Islander at 0.3 (Fig. 2). For the NH White cohort, the APC increased at a rate of 0.7% per year from 1999 to 2013, after which it decreased at a rate of -6.1% annually until 2017, and then − 1.3% annually until 2020. For the NH Black cohort, the APC decreased at a rate of -2.3% per year between 1999 and 2020, the steepest of any group. In NH Asian or Pacific Islanders and Hispanics, the APC decreased at a rate of -1.2% annually.

**Fig. 3:**
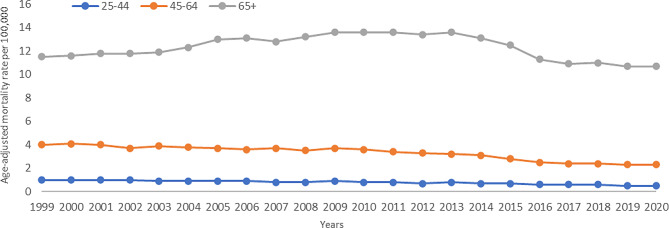
Trends in age-adjusted, melanoma‐related mortality rates stratified by age in the United States, 1999 to 2020. Ages 25–44: 1999–2013 APC − 2.3* (95% CI -2.7 to -0.8), 2013–2020 APC − 5.7* (95% CI -9.6 to -4.0); Ages 45–64: 1999–2013 APC − 1.4* (95% CI -1.8 to -0.9), 2013–2016 APC − 9.0* (95% CI -10.7 to -2.2), 2016–2020 APC − 2.4 (95% CI -4.9 to 2.6); Age > 65: 1999–2010 APC 1.7* (95% CI 1.4 to 2.3), 2010–2014 APC − 0.9 (95% CI -2.2 to 1.0), 2014–2017 APC − 6.4* (95% CI -7.6 to -4.6), 2017–2020 APC − 0.4 (95% CI -2.0 to 2.6) *Indicates that the annual percentage change (APC) is significantly different from 0 at α = 0.05

Between 1999 and 2020, individuals aged 65 + had the highest AAMR at 12.2, followed by those aged 45–64 years at 3.3, and then 25–44 years at 0.8 (Fig. 3). For the 65 + cohort, the melanoma death rates rose at 1.4% per year from 1999 to 2013, after which they steeply fell at a rate of -6.1% annually until 2017, where the slope flattened to -0.5% annually until 2020. In individuals aged 45–64, the APC decreased at a rate of -1.4% annually from 1999 to 2013, and then steeply fell at a rate of -9.0% per year until 2016, after which the slope flattened to -2.4% until 2020. For individuals aged 25–44, the APC decreased at a rate of -2.1% per year from 1999 to 2013, and between 2013 and 2020 the APC decreased at a rate of -5.7% annually.

**Fig. 4:**
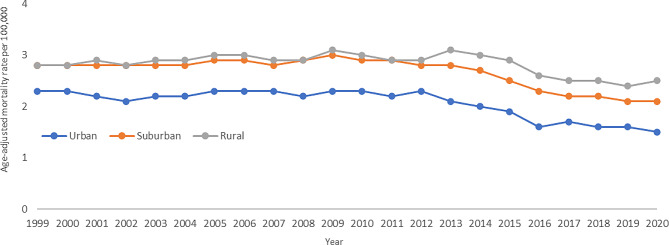
Trends in age-adjusted, melanoma‐related mortality rates stratified by geographic density in the United States, 1999 to 2020. Urban: 1999–2012 APC 0.2 (95% CI -1.1 to 1.4), 2012–2016 APC − 7.1 (95% CI -9.8 to 1.3), 2016–2020 APC − 2.6 (95% CI -5.2 to 2.2); Suburban: 1999–2010 APC 0.5* (95% CI 0.2 to 1.2), 2010–2014 APC − 2.0 (95% CI -3.1 to 0.1), 2014–2017 APC − 6.8* (95% CI -8.0 to -5.0), 2017–2020 APC − 1.4 (95% CI -3.0 to 1.7); Rural: 1999–2014 APC 0.5 (95% CI -0.6 to 1.5), 2014–2017 APC − 6.5 (95% CI -8.5 to 1.6), 2017–2020 APC − 0.2 (95% CI -4.0 to 4.9) *Indicates that the annual percentage change (APC) is significantly different from 0 at α = 0.05

Between 1999 and 2020, rural populations experienced the highest AAMR at 2.8, followed by suburban populations at 2.6, and then urban populations at 2.1 (Fig. 4). In urban populations, the melanoma death rates remained stable between 1999 and 2011 at a 0.2% annual rise. After 2011, the melanoma death rates began to fall at a rate of -4.8% annually until 2020. For suburban populations, the APC again remained stable between 1999 and 2013 at a 0.2% annual increase. Between 2013 and 2016, the APC decreased at a rate of -7.5% per year, and between 2016 and 2020 the APC decreased at a rate of -2.4% per year. In rural populations, the APC decreased from 1999 to 2014 at a rate of 0.5% annually, and then between 2014 and 2017 at a rate of -6.6% annually. After 2018, the melanoma death rate decreased at a slower rate of -0.2% annually.

**Fig. 5:**
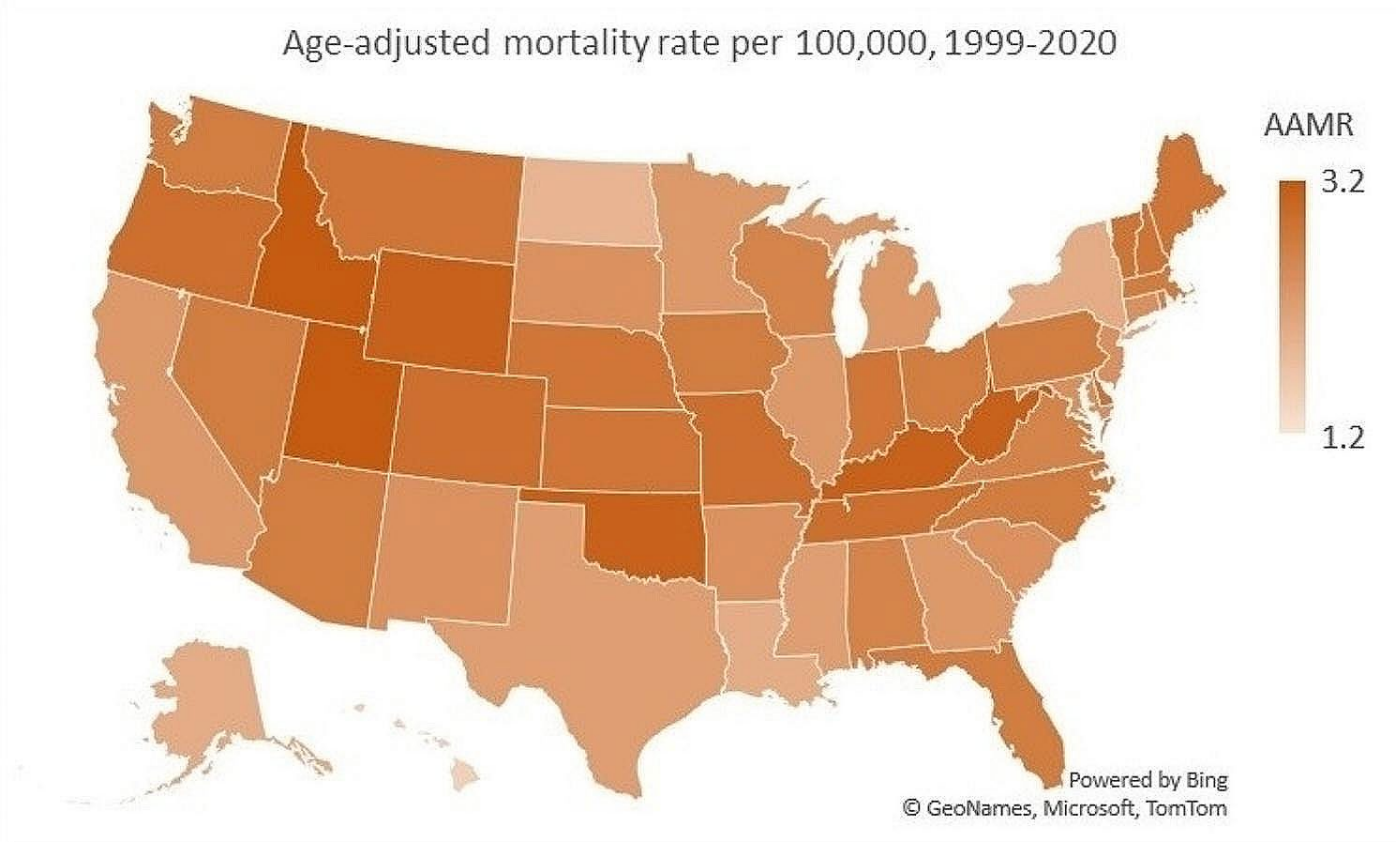
State-level, age‐adjusted, melanoma‐related mortality rates in the United States from 1999 to 2020

Melanoma death rates were roughly stable between all US Census Regions (Fig. 5). Between 1999 and 2020, the South experienced the steepest decrease in melanoma death rates at -1.6% annually, whereas the Midwest experienced the slowest decrease at a rate of -0.6% annually. States in the 90th percentile of melanoma mortality included Idaho, Utah, and Oklahoma, whereas states in the 10th percentile included Alaska, Louisiana, and North Dakota.

## Discussion

Our study reports notable trends in the mortality rate of melanoma in the United States between 1999 and 2020. First, the mortality rate was mostly stable until 2013, after which it began to fall at a rate of 6.6% annually. Second, men had a mortality almost twice that of women in 1999; however, these differences narrowed over time. Third, NH White individuals experienced the highest mortality at 3.2, almost five times that of Hispanics, who had the next highest mortality at 0.7. Fourth, elderly individuals experienced the highest mortality of any age group at 12.2, with the sharpest decrease at 6.1% annually. Fifth, rural populations had the highest mortality rates, with all regions of the United States noticing similar mortality rates and trends over time.

There are several reasons why the incidence of melanoma increased while mortality rate steadily declined since 2013. Prior to the year 2000, melanoma was initially diagnosed through unaided visual skin examination followed by biopsy for confirmation. After the year 2000, dermoscopy was routinely implemented into the clinical setting resulting in improved sensitivity and specificity. A dermoscope is a tool that noninvasively visualizes color and structure patterns within the layers of the skin at the site of the lesion—allowing dermatologists to more easily and accurately differentiate melanoma from other nevi without the pain, delay, and cost associated with unaided visual inspection and biopsy [13–16]. Dermoscopy significantly improved sensitivity for diagnosing melanoma, often avoiding the need for unnecessary skin biopsy [17]. Both dermatologists and family practitioners have become trained to use dermoscopy, resulting in reduced biopsies resulting in a reduction in unwarranted biopsies for non-melanoma diagnoses and a significant reduction in the size of nevus needed to detect melanoma [13, 14].

The improved sensitivity that dermoscopy provided in early detection resulted in an increased incidence of cutaneous melanoma due to the ability to diagnose melanoma in situ (MIS) rather than malignant melanoma (MM). Some argue that dermoscopy contributed to a “false epidemic,” of dangerous melanoma by detecting thinner tumors with less metastatic potential—an artifact of improved diagnostic scrutiny rather than carcinogenesis. Indeed, most studies analyzing melanoma incidence between the 1990s and 2000s reported higher annual percentage increases of MIS and thin MM relative to thick MM. However, a true increase in thick MM incidence was observed in a US SEER database study between 1992 and 2004, particularly in low socioeconomic status (SES) groups [18]. This suggests that melanoma incidence cannot be blamed on early detection of thinner tumors alone. An Italian study from 2012 − 2017 showed that decreasing tumor thickness at the time of diagnosis contributed to improved 5-year survival. Dermoscopy has been shown to improve accuracy of cutaneous melanoma detection compared to the naked eye, but it does not fully explain the mortality trends in our study [14, 19, 20].

Providers and policymakers adopted prevention and early detection strategies to decrease melanoma incidence and mortality. For example, indoor tanning, and the associated ultraviolet (UV) radiation, was officially classified as a human carcinogen in 2009 by the World Health Organization International Agency for Research on Cancer (IARC) [21]. Increased public knowledge of its harmful effects and restrictive legislation in some countries has led to decreased global prevalence of indoor tanning in adolescents and adults. A meta-analysis of worldwide indoor tanning prevalence from 1986 to 2012 found a past-year prevalence of 18% and 14% among adolescents and adults, respectively [22]. From 2013 to 2018, another meta-analysis by Rodriquez-Acevedo et al. showed a 70% reduction in adolescent use (6.7% prevalence) and 35% reduction in adult use (11.9% prevalence) compared to the previous study [23]. In this study’s North American population specifically, the prevalence of tanning indoors from 2009 to 2018 was 7.6% in adolescents and 14.4% in adults, which showed a significant decrease in sunbed use in adolescents but not adults in the 1986 to 2012 population. Another 2020 analysis using the Health Information National Trend Survey from 2007 to 2018 reported a significant decrease in U.S. indoor tanning prevalence from 2007 (10%) to 2018 (4%) [24]. States with the greatest decreases in indoor tanning prevalence had adopted youth access legislation such as parental permission requirements and outright prohibition for minors. Age restriction was associated with 45.7% lower indoor tanning prevalence among female U.S. high school students, a population targeted by national CDC campaigns (“Burning Truth”) in 2014 that used social media platforms to target young female audiences. The steep decline of AAMR from 2013 to 2017 in our data seemed to coincide with national efforts to raise awareness of UV exposure risk. However, the correlation between exposure and mortality may be misleading. The greatest reductions in melanoma mortality in our study occurred in men, adults aged > 65, and African Americans, whereas the greatest decrease in indoor tanning prevalence reported by previous meta-analyses occurred in younger, Caucasian women. Additionally, incidence of melanoma in the U.S. between 2009 and 2018 had increased from a total rate per 100,000 population of 20.4 to 22.0, affecting primarily adults over the age of 55 [25]. Melanoma incidence in U.S. adolescents and young adults aged 15–24 fell steeply in this period, with an APC − 4.9, however mortality trends in this population were not included in our study. Overall, the improvement in rates of melanoma mortality do not seem to be associated with lower incidence or risk factor modification.

Educational programs on children’s knowledge and behavior towards the sun may contribute to decreased melanoma incidence and mortality, as recently seen in indoor tanning. One study evaluated the impact of a targeted digital educational program on melanoma knowledge and behaviors, finding that their educational program led to a 22% increase in melanoma knowledge and a decrease in sun-protective behaviors [26]. Another study evaluated the US Environmental Protection Agency’s SunWise School Program, an environmental education program for sun safety of children in primary and secondary school [27]. They found a significant improvement in multiple knowledge variables, including appropriate type of sunscreen to be used for outdoor play, highest UV Index number, and need for hats and shirts outside. Wu and colleagues designed an educational intervention called the Melanoma Education and Risk Information Team (MERIT). MERIT emphasizes the mechanisms and genetic risk factors that lead to melanoma development and is designed to be delivered to children at risk of the disease due to family history [28]. These results emphasize the potentially profound impact of a low-cost, convenient educational intervention on decreasing melanoma incidence and mortality rates.

Our data demonstrate a significant decrease in melanoma deaths from 2013 to 2017 and then a slower decrease after 2017 across most groups. In addition to the increased use of dermoscopy and efforts by governing bodies to increase awareness about the harmful effects of UV radiation, there was increased usage latest generation therapies, such as immune checkpoint inhibitors, and decreased usage of cytokines and chemotherapy from 2011 to 2016 [29]. These changes largely impact advanced or inoperable melanomas, which represent the main source of mortality. Ipilimumab was approved for melanoma in 2013 and functions as an anti-CTLA-4 monoclonal antibody blocking downstream signaling and resulting in a more robust and prolonged T-cell-mediated immunity against melanoma tumor cells [30]. When compared to treatment with chemotherapy alone, 4-year survival for patients treated with ipilimumab was between 13.8 and 28.4%, and a positive trend was seen in survival rates in patients who were given higher doses of ipilimumab [31]. One meta-analysis including close to 5,000 patients with advanced melanoma treated with ipilimumab showed that almost 20% of alive at 10 years, contributing to decreases in annual mortality seen in our study [32]. Two PD1 blockades, nivolumab, and pembrolizumab, have been approved by the FDA for use in patients with metastatic melanoma and BRAF-mutated melanoma [33]. PD-L1 is highly expressed on a variety of cancer cells, including melanoma, and helps these cells evade immune detection when it binds to PD1 receptors [34, 35]. PDL1 overexpression by cancer has been shown to limit T cell activity; whereas, limiting PDL1 expression allows T cells to assist in an immune response to cancer tumors [36]. These same mutations have been demonstrated in advanced cutaneous squamous cell carcinomas, rendering them vulnerable to treatment with therapies like nivolumab, cemiplimab, and pembrolizumab [37]. Combination immunotherapy has shown promise in reaching durable clinical responses in patients with advanced melanoma. The CheckMate 067 Trial compared the use of nivolumab alone and with ipilimumab and found that the combination of both drugs together had a higher median overall survival rate [38]. A number of ongoing large studies are being conducted to examine the ideal treatment sequence, including ECOG 6134, a randomized phase III trial comparing ipilimumab plus nivolumab followed by dabrafenib plus trametinib versus dabrafenib plus trametinib followed by ipilimumab and nivolumab [39]. Considering our data and these studies, the steep decline in melanoma deaths shown in our data may be strongly associated with the approval and incorporation of these novel drugs into treatment.

Results from our study indicate a decrease in melanoma deaths experienced across the country; however, when analyzed geographically, the South had the largest decrease in their melanoma death rate compared to other regions in the country. In addition to these findings, it was seen that those from rural populations had experienced a higher mortality compared to suburban and urban populations. These findings are indicative of a possible association between location and access to routine healthcare facilities and melanoma mortality risk. In a prospective cohort study performed by Qureshi et al. from 1984 to 2002, they examined the risk of melanoma and UV index, a linear measure of UV radiation intensity. Their results demonstrated that the risk of melanoma was significantly higher in women who lived in regions with a UV index of 6 from birth to 15 years of age [40]. When taken with the results from our study, this suggests a link between geographic region and risk for melanoma. In addition to higher UV indexes in southern states, another reason for this difference in geographic melanoma mortality geographically may be due to differences in screening practices. In a study by Fernandez et al., differences in Florida skin cancer screening rates with various US regions were compared using the National Health Interview Survey from 2000 to 2005 [41]. They found that individuals from Florida who identified as a 70 + year old female, a non-Hispanic ethnicity, a graduate from high school, healthcare insured, or having a service industry job or facing unemployment had higher skin cancer screening rates than those across the rest of the US [41]. Thus, the geographic variation in melanoma mortality seen in our study may be tied to the increased screening rates for skin cancer in the South. One could also speculate that there are differences in the make-up of melanoma incidence by stage by geographic region. For example, regions with lower screening may have higher incidence of late-stage disease. However, no study published to date has explored this hypothesis. Increased adoption of teledermatology may improve screening and mortality in rural areas. A mobile phone application developed for skin cancer screening demonstrated similar sensitivity in detecting malignancies compared with face-to-face evaluation in the clinic, which may be considered as an ancillary option for rural communities with limited access to dermatology clinics [42].

Our study is not without limitations. For one, the CDC WONDER database is linked to individual death certificates, which could potentially be miscoded. However, the CDC WONDER database has been widely used in studies assessing cancer and other causes of mortality and undergoes internal validation and quality assurance measures which limits the potential for misclassification [8, 9, 43]. Next, because the CDC WONDER database uses data collected from death certificates, it is possible that mortality may be overestimated due to occult cases of melanoma reported on death certificates [44]. Additionally, the database does not report data on important variables that may impact mortality, including histologic subtype, Breslow thickness, stage, treatments received, duration of therapy, and various socioeconomic factors including income. Despite these limitations, we opted to use the CDC WONDER database because it contains geographic data not reported elsewhere. Finally, the changes in the epidemiology and mortality of melanoma are the result of a complex interplay of phenomena, ranging from improved diagnosed techniques to increased attention to appearance or cultural trends. Thus, it is difficult, if not impossible, to determine the exact nature of what modifies and influences these data.

## Conclusion

Our study demonstrates a significant improvement in melanoma mortality in the United States with the sharpest decrease appearing after 2013. This may be related to several factors, including increased use of dermoscopy, widespread adoption of immune checkpoint inhibitors, and extensive education regarding the dangers of excessive UV light exposure. Further, our study revealed disparities in mortality rates among rural populations when compared with urban and suburban groups. The clinical implication of this should stimulate the development educational interventions toward vulnerable subgroups, such as rural populations, in order to increase melanoma screening rates and improve sun-protective behaviors.

## Data Availability

The data used for this study are publicly available at https://wonder.cdc.gov.
